# Inhibiting PP2A Upregulates B7-H3 Expression and Potentially Increases the Sensitivity of Malignant Meningiomas to Immunotherapy by Proteomics

**DOI:** 10.3389/pore.2022.1610572

**Published:** 2022-09-20

**Authors:** Boyi Hu, Shuyu Hao, Yazhou Miao, Yuxuan Deng, Jing Wang, Hong Wan, Shaodong Zhang, Nan Ji, Jie Feng

**Affiliations:** ^1^ Beijing Neurosurgical Institute, Capital Medical University, Beijing, China; ^2^ Department of Neurosurgery, Beijing Tiantan Hospital, Capital Medical University, Beijing, China

**Keywords:** immune checkpoint, malignant meningiomas, PP2A inhibitor, B7-H3, tumor proliferation

## Abstract

Malignant meningiomas have a high mortality rate and short survival time and currently have no effective treatment. In our study, proteomics analysis was performed to identify highly expressed proteins as therapeutic targets in malignant meningiomas. Cell Counting Kit-8 (CCK-8) assays were performed to verify the effect of LB-100 on the growth of malignant meningiomas. In addition, immunoblotting was used to verify the expression of B7-H3 and phosphorylation of STAT1 (Tyr701) in tissues and cells. Our results show that STAT1 and CD276 (B7-H3) regulated by PP2A were enriched in GO_IMMUNE_EFFECTOR_PROCESS and GO_REGULATION_OF_IMMUNE_SYSTEM_PROCESS. The immunotherapy target protein B7-H3 was confirmed to be upregulated in malignant meningiomas compared with meningothelial (*p* = 0.0001) and fibroblastic (*p* = 0.0046) meningiomas. *In vitro*, the PP2A inhibitor LB-100 suppressed the growth and invasion of malignant meningioma cells. Notably, the PP2A inhibitor LB-100 increased the phosphorylation of STAT1, thereby increasing the expression of the immune checkpoint protein B7-H3 in malignant meningioma cells *in vitro*. In conclusion, B7-H3 was found to be upregulated in malignant meningiomas. The PP2A inhibitor LB-100 increased the phosphorylation of STAT1 and B7-H3 expression, which could increase the sensitivity of malignant meningiomas to B7-H3 targeted immunotherapy.

## Introduction

Meningiomas are the most common type of primary central nervous system (CNS) tumor, accounting for approximately 38.4% of all CNS tumors [1]. Malignant meningiomas (WHO grade 2 and 3) account for 15%–20% of all meningiomas [2]. Malignant meningiomas have a greater possibility of recurrence and aggressive growth and a poorer prognosis. At present, there is no effective treatment for malignant meningiomas.

Recently, an increasing number of studies have focused on protein phosphatase 2A (PP2A). PP2A is involved in cell signal transduction and plays an important role in the process of mitosis and apoptosis [3, 4]. PP2A is a serine/threonine phosphatase composed of three subunits: A, B, and C. The A and C subunits play a structural and catalytic role, and the B subunit plays a regulatory role. PP2A inhibitors can inhibit the growth of various tumors. LB-100 is a small molecule inhibitor of PP2A that can bind to the C subunit and inhibit the catalytic activity of PP2A. LB-100 can penetrate the blood–brain barrier (BBB) and has potential clinical application value [5].

It is widely known that signal transducers and activators of transcription (STATs) are one of the transcription factors regulated by PP2A. The STAT family is composed of 7 subtypes: STAT1, STAT2, STAT3, STAT4, STAT5, STAT6 and STAT7. STATs are activated by phosphorylation and transferred to the nucleus to regulate the expression of target genes and mediate various biological processes, such as tumor immune escape [6]. Studies have shown that STATs regulate the expression of the immune checkpoint protein programmed cell death ligand 1 (PD-L1) in tumor cells. In lung adenocarcinoma cells, IFN-γ can induce the activation of the JAK2-STAT1 pathway, which in turn increases the expression of the immunosuppressive checkpoint PD-L1 and enables tumor immune escape [7]. In hepatocellular carcinoma, nicotinamide phosphoribosyltransferase (NAMPT) can also increase the expression of PD-L1 by promoting the phosphorylation of STAT1, thereby mediating the immune escape of liver cancer [8]. In melanoma, renal cell carcinoma, head and neck squamous cell carcinoma and non-small-cell lung cancer, Chen et al. found that STAT1 can mediate IFN-γ-induced PD-L1 expression on tumor cells [9].

Immune checkpoint proteins as targets of immunotherapy are one of the current research hotspots. As a recently discovered immune checkpoint protein, B7-H3 (also known as CD276) has become a potential target for tumor immunotherapy [10]. Studies have shown that the overexpression of B7-H3 in various tumor tissues is associated with tumor metastasis, invasion and poor prognosis [11, 12, 13, 14]. More importantly, B7-H3 plays an important role in the immune escape of tumors. Purvis et al. confirmed that the activation of STAT1 can downregulate the expression of MYC in medulloblastoma cells and then downregulate the expression of B7-H3 to exert its antitumor effect [15]. However, whether the PP2A inhibitor LB-100 can affect the expression of B7-H3 by regulating STAT1 has not yet been reported. At present, B7-H3 has been used as a target in the immunotherapy of various malignant tumors. For example, anti-B7-H3 monoclonal antibodies can significantly inhibit the growth of renal cell carcinoma and bladder cancer [16]; chimeric antigen receptor (CAR) T cells targeting B7-H3 have a significant therapeutic effect on malignant meningiomas [17]; and an antibody–drug conjugate targeting B7-H3 has obvious antitumor activity against malignant tumors such as breast cancer and melanoma [18]. Therefore, how to increase the expression of B7-H3 in tumors and promote the sensitivity of tumors to B7-H3 targeted therapy is also an important scientific problem for tumor immunotherapy targeting B7-H3. Whether the PP2A inhibitor LB-100 can upregulate the expression of B7-H3 and in turn affect the sensitivity of malignant meningiomas to B7-H3 targeted immunotherapy has not been reported.

Proteomics can not only study all proteins in tumors on a large scale but also systematically detect protein–protein interactions, protein networks, regulation and functions of signaling pathways. In this study, we used proteomics to systematically identify immune-related proteins and their signal regulatory pathways in malignant meningiomas to provide new potential targets for the treatment of malignant meningiomas. Our study will also explore the B7-H3 immune checkpoint protein signaling pathway in malignant meningiomas. Meanwhile, we will clarify whether the PP2A inhibitor LB-100 upregulates the expression of B7-H3 by increasing the phosphorylation of STAT1, which may increase the sensitivity of malignant meningiomas to B7-H3-targeted immunotherapy.

## Materials and Methods

### Patients and Specimens

All thirteen tumor samples were obtained surgically at Beijing Tiantan Hospital. Fresh tumor samples from these patients were frozen and stored in liquid nitrogen. Thirteen tumor samples were classified according to the 2016 WHO classification. Thirteen tumor samples included six cases of meningothelial meningiomas, three cases of fibroblastic meningiomas and four cases of malignant meningiomas. The four malignant meningiomas were anaplastic meningiomas (WHO grade 3). In this study, four malignant and six meningothelial meningiomas were used for the proteomic analysis and Western blot analysis, and three fibroblastic meningiomas were used for Western blot analysis.

This study was approved by the Ethics Committee of Beijing Tiantan Hospital (KY2021-158-01). Informed consent was obtained from all enrolled subjects, and this study was performed in accordance with the Declaration of Helsinki.

### Protein Preparation and Proteomics Analysis

**FIGURE 1:**
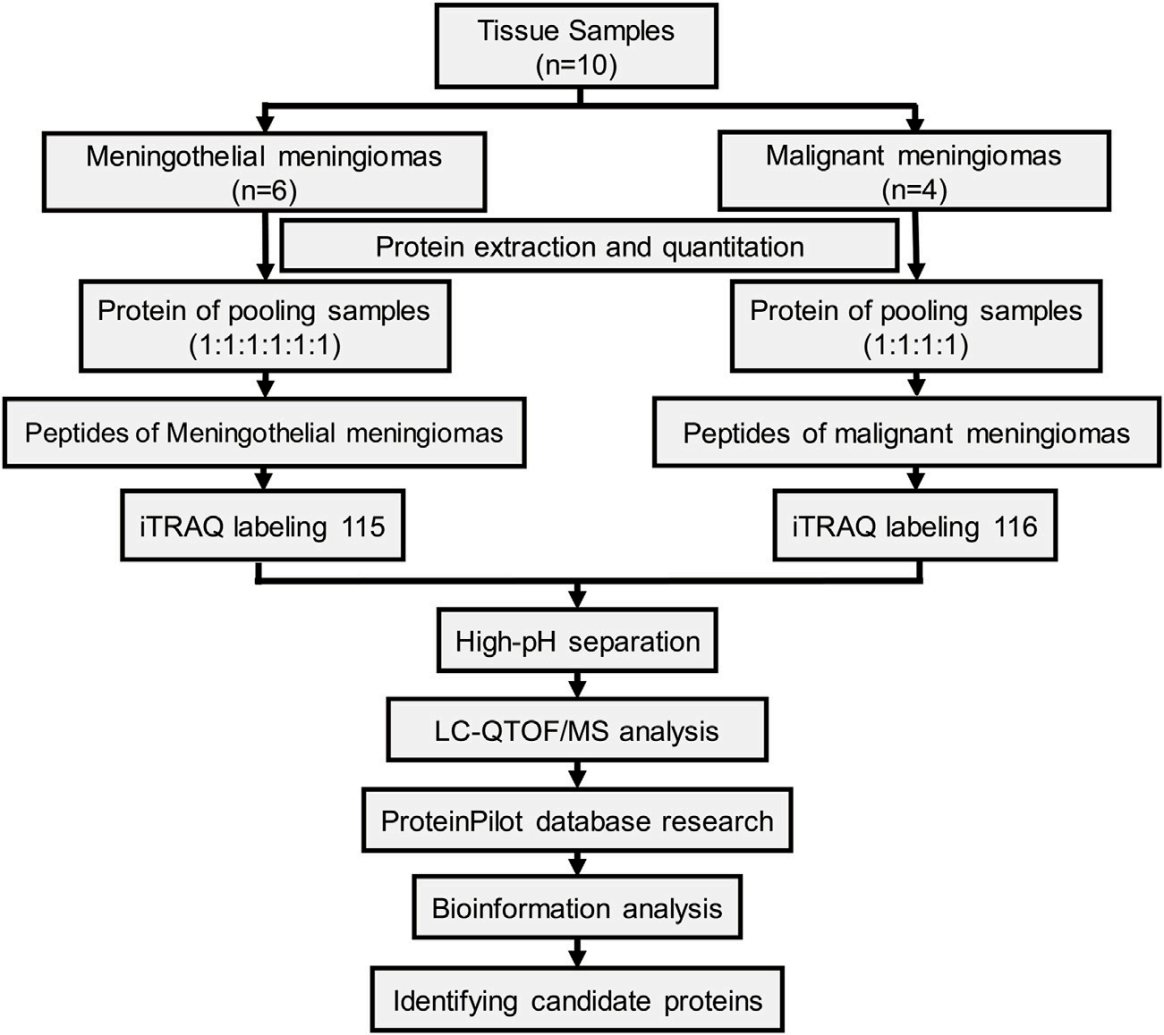
Workflow of the proteomic strategy.

The workflow of the protein preparation and protein profiling are shown in Figure 1. Meningioma tissues were divided into two groups according to pathological subtypes. Proteins from meningiomas were extracted using nondenaturing lysis buffer (Applygen C1050, Beijing, China) with 1% Protease Inhibitor Cocktail (Beyotime, Shanghai, China) and 1% Phosphatase Inhibitor Cocktail (Epizyme, Shanghai, China). Protein concentrations were determined using the BCA Protein Assay Kit (Pierce, Rockford, IL 61101 United States).

The proteins from all six meningothelial meningiomas or four malignant meningiomas were equally combined into a single pool. A mass of 200 μg of each pooled sample was denatured, reduced and alkylated as described in the iTRAQ protocol (Applied Biosystems) and digested overnight with 0.1 μg/μL trypsin solution at 37°C. Pooled digested meningothelial and malignant meningioma samples were labeled with 115 and 116 iTRAQ tags, respectively, according to the manufacturer’s protocol (Ab Sciex). The tagged peptides were dried via vacuum centrifugation and combined in one tube. Strong cation exchange (SCX) chromatography was performed according to a previously described method [19]. Briefly, the pooled sample was separated on an Apoly LC SCX column (4.6 × 250 mm, 5 μm, 100 Å) using a Nexera XR instrument (Shimadzu, Japan), and the labeled peptides were detected by ultraviolet radiation using an SPD-20A (Shimadzu, Japan). In this study, a total of 55 fractions were collected, dried by speed vacuum centrifugation, and combined into 12 fractions according to the SCX chromatogram. Each fraction was injected onto a desalting column (10 × 0.3 mm, 5 μm-C18, 120 Å) and separated on an analytical column (0.3 × 150 mm, 3 μm C18, 120 Å) using an Eksigent micro-LC instrument (Eksigent, Dublin, CA, United States). The samples separated *via* capillary high-performance liquid chromatography were subsequently analyzed using a Triple TOF 5600+ system (Ab Sciex, United States).

Protein identification and differential expression were performed using the ProteinPilot software package (version 5.0, Applied Biosystems Sciex, United States) and searched against the SwissProt database (March 2020). The following search parameters were utilized to analyze the QTOF/MS data: trypsin as the digestion enzyme, with a maximum of two missed cleavages allowed; fixed modifications of carbamidomethyl (C) and iTRAQ Plex (K and N-terminus); variable modifications of oxidation (M); peptide mass tolerance of ±20 ppm; fragment mass tolerance of ±0.1 Da; and peptide FDR ≤0.01.

### Cell Culture and Materials

The IOMM-Lee human meningioma cell line was purchased from the American Type Culture Collection (ATCC, Rockville, MD, United States) and cultured in Dulbecco’s modified Eagle’s medium (DMEM, Biosharp, China) supplemented with 10% fetal bovine serum (Gibco, Auckland, United States) in a humidified incubator at 37°C with 5% CO_2_.

LB-100 was provided by Selleck Chemicals (Cat. No. S7537, United States). A stock solution of LB-100 in 50 mM DNase/RNase-Free Distilled Water (Cat. No. 10977015, Thermo Fisher, United States) stored at −80°C was diluted as needed in cold phosphate buffered saline (PBS; Biosharp, China).

### Cell Viability Assay

Cell viability was assessed by Cell Counting Kit-8 (CCK-8) assay (Biosharp, China). Ninety-six-well plates were seeded with approximately 3 × 10^3^ IOMM-Lee cells per well. The cells were treated with various concentrations of LB-100 (10, 20, 40, and 80 uM). The CCK-8 assays were carried out according to the manufacturer’s instructions after 24, 48, and 72 h of treatment. Absorbance values were determined at 450 nm on a Synergy H1 MFD spectrophotometer (BioTek). CCK-8 assays were performed in quintuplicate.

### Cell Protein Extraction and Western Blotting Analysis

Whole-cell protein was obtained by applying nondenaturing lysis buffer (C1050; Applygen, Beijing, China) with 1% Protease Inhibitor Cocktail for general use (Beyotime, Shanghai, China) and 1% Phosphatase Inhibitor Cocktail (Epizyme, Shanghai, China), and then the lysate was purified through centrifugation. The BCA Protein Assay Kit (Pierce, Rockford, IL 61101 United States) was used to quantify the protein in the supernatant. Equal amounts of protein were added to 25% 5× loading buffer and then denatured at 100°C for 5 min. Soluble proteins (20 μg) were separated with the BioSciTM NewFlash Protein AnyKD PAGE Kit (Dakewe, China), transferred to polyvinylidene fluoride (PVDF) membranes (Cat No. IPVH00010, Merck Millipore Ltd., United States), incubated with NcmBlot blocking buffer (Cat No. P30500, NCM, China) for 10 min and then washed twice with Tris-buffered saline/Tween-20 (TBST). Then, the membranes were probed overnight with the corresponding primary antibody at 4°C followed by three 10-min washes with TBST. Subsequently, the membranes were incubated with secondary antibodies conjugated to horseradish peroxidase at room temperature for 1 h followed by three 10-min washes with TBST. Blots were visualized by enhanced chemiluminescence, and densitometry was performed on an Amersham Imager 600 (GE). Rabbit anti-STAT1 antibody (Cat. #14994S, dilution factor 1:500, CST), mouse anti-STAT1 (phospho Y701) antibody (Cat. ab29045, dilution factor 1:1000, Abcam) and rabbit anti-B7-H3 antibody (Cat. #14058S, dilution factor 1:500, CST) were used for Western blot analysis. All assays were performed three times. The final data were subjected to grayscale scanning and semiquantitative analysis using ImageJ software (https://imagej.nih.gov/ij/download.html).

### Statistical Analysis

Statistical analysis was performed on results from at least 3 independent replicates. All data are presented as the mean ± standard error of the mean (SEM). One-way ANOVA was used for comparisons between more than two groups, and post-hoc Tukey’s multiple comparisons test was used for multiple comparisons. A *p* value less than 0.05 was considered statistically significant.

## Results

### Protein Profiling of Meningothelial and Malignant Meningiomas

We performed a quantitative proteomics analysis to determine the differences between meningothelial and malignant meningiomas. A total of 985839 unique peptides mapped to 5581 proteins were identified according to the ProteinPilot database, and 305 proteins were considered to be significantly differentially expressed (PVal <0.05, fold change >2 or <0.5) between meningothelial meningiomas and malignant meningiomas (Supplementary Table S1). Among these proteins, 147 were upregulated in malignant meningiomas, and 158 were downregulated in malignant meningiomas.

### Enrichment Analysis

**FIGURE 2:**
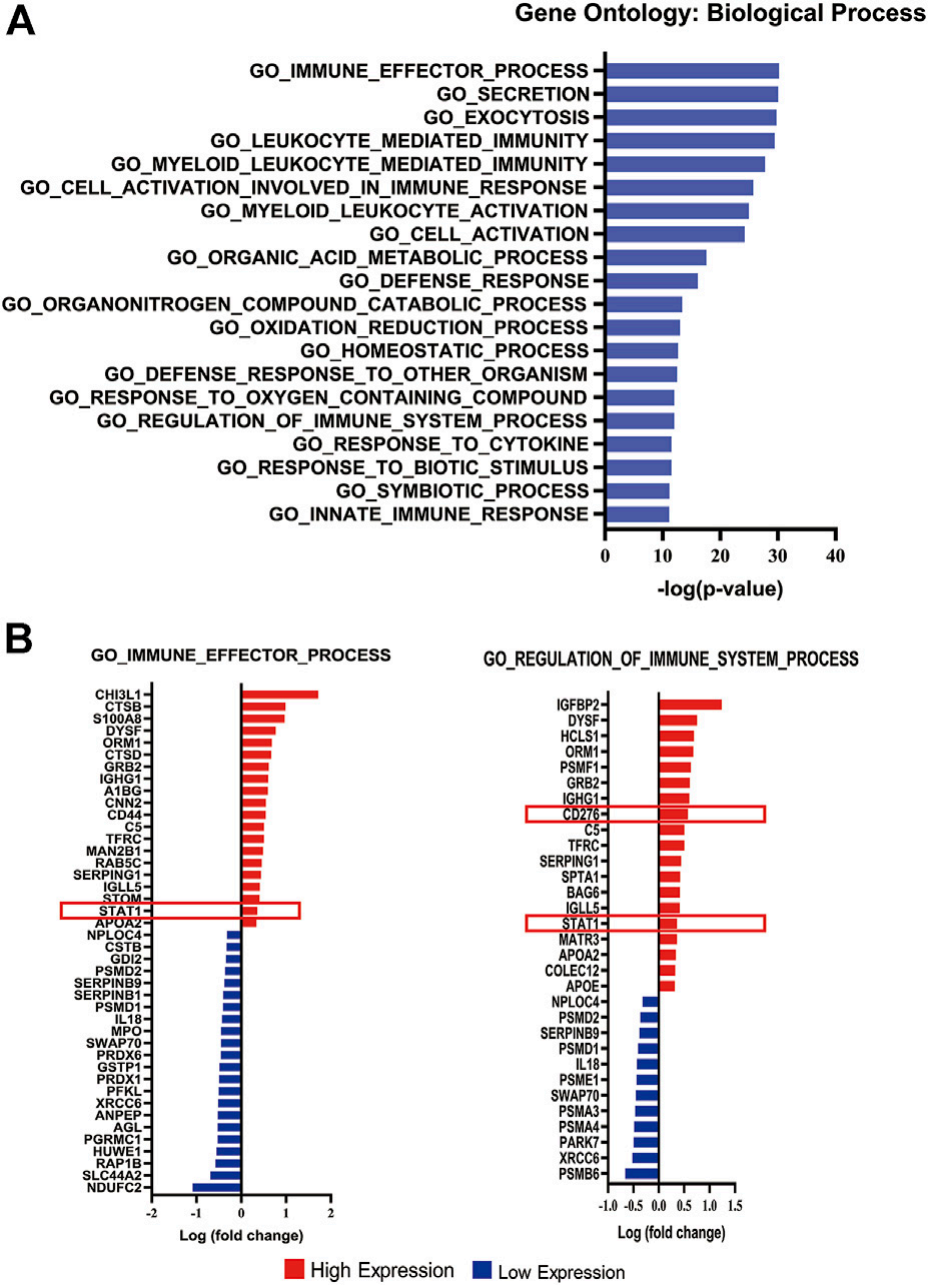
Enrichment analysis of differentially expressed proteins. **(A)** Bar chart displaying the top 20 significant pathways according to the enrichment analysis. **(B)** Diagram listing the proteins involved in the GO_IMMUNE_EFFECTOR_PROCESS and GO REGULATION OF IMMUNE SYSTEM PROCESS pathways.The X-axis represents the log(fold change). Red bars represent upregulated expression, and blue bars represent downregulated expression in malignant meningiomas. The red box displays the upregulated expression of STAT1 and B7-H3 in malignant meningiomas.

We performed enrichment analysis based on the biological process of gene ontology at the Gene Set Enrichment Analysis (GSEA) website (http://www.gsea-msigdb.org/gsea/msigdb/annotate.jsp). The top twenty biological processes (*p* < 0.05) are shown in Figure 2A. According to the enrichment analysis, we found that among all immune-related biological process pathways, STAT1 and CD276 (B7-H3), which were regulated by PP2A [15, 20], were enriched in GO_IMMUNE_EFFECTOR_PROCESS (−log p value = 30.18) and GO_REGULATION_OF_IMMUNE_SYSTEM_PROCESS (−log p value = 12.00). The complete biological process diagram is shown in Supplementary Table S2.

Of forty-two differentially expressed proteins enriched in GO_IMMUNE_EFFECTOR_PROCESS, twenty exhibited higher expression levels, and twenty-two exhibited lower expression levels in malignant meningiomas (Supplementary Table S3). Additionally, of the thirty-one differentially expressed proteins enriched in GO_REGULATION_OF_IMMUNE_SYSTEM_PROCESS, nineteen exhibited higher expression levels, and twelve exhibited lower expression levels in malignant meningiomas (Supplementary Table S4). STAT1 and CD276 were highly expressed in malignant meningiomas compared with meningothelial meningiomas (Figure 2B).

### Validating the Overexpression of B7-H3 in Malignant Meningiomas

**FIGURE 3:**
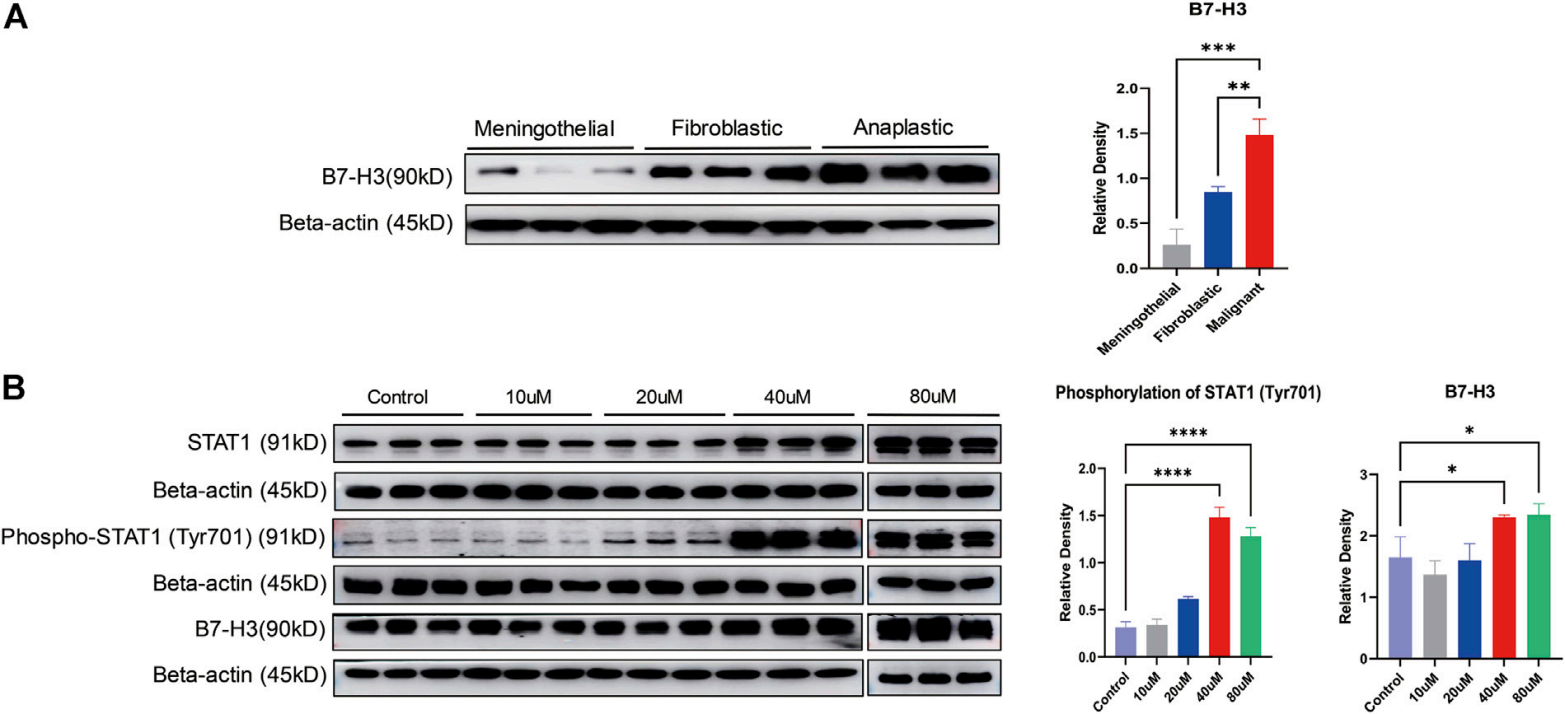
Validation of the expression of B7-H3 and STAT1 in meningiomas. **(A)** The expression of B7-H3 is displayed in different subtypes of meningiomas. The bar chart shows the relative density of B7-H3. The X axis marks the subtypes, and the Y axis represents the relative density; **p < 0.01, ***p < 0.001. **(B)** The expression of STAT1, phospho-STAT1 (Tyr701) and B7-H3 is displayed at different concentrations of LB-100 (10, 20, 40, and 80 uM). Two bar charts show the phosphorylation of STAT1 (Tyr701) and the relative density of B7-H3. The X axis marks different concentrations of LB-100, and the Y axis represents the relative density; *p < 0.05, ****p < 0.0001.

To further confirm the findings in the proteomic experiments, Western blot analysis was performed to validate the expression of B7-H3 among meningothelial, fibroblastic and malignant meningiomas. There was a significant difference in B7-H3 expression among the different subtypes (*p* = 0.0002). B7-H3 was significantly overexpressed in malignant meningiomas compared with meningothelial (*p* = 0.0001) and fibroblastic (*p* = 0.0046) meningiomas (Figure 3A).

### LB-100 Inhibits the Proliferation of Malignant Meningioma Cells

**FIGURE 4:**
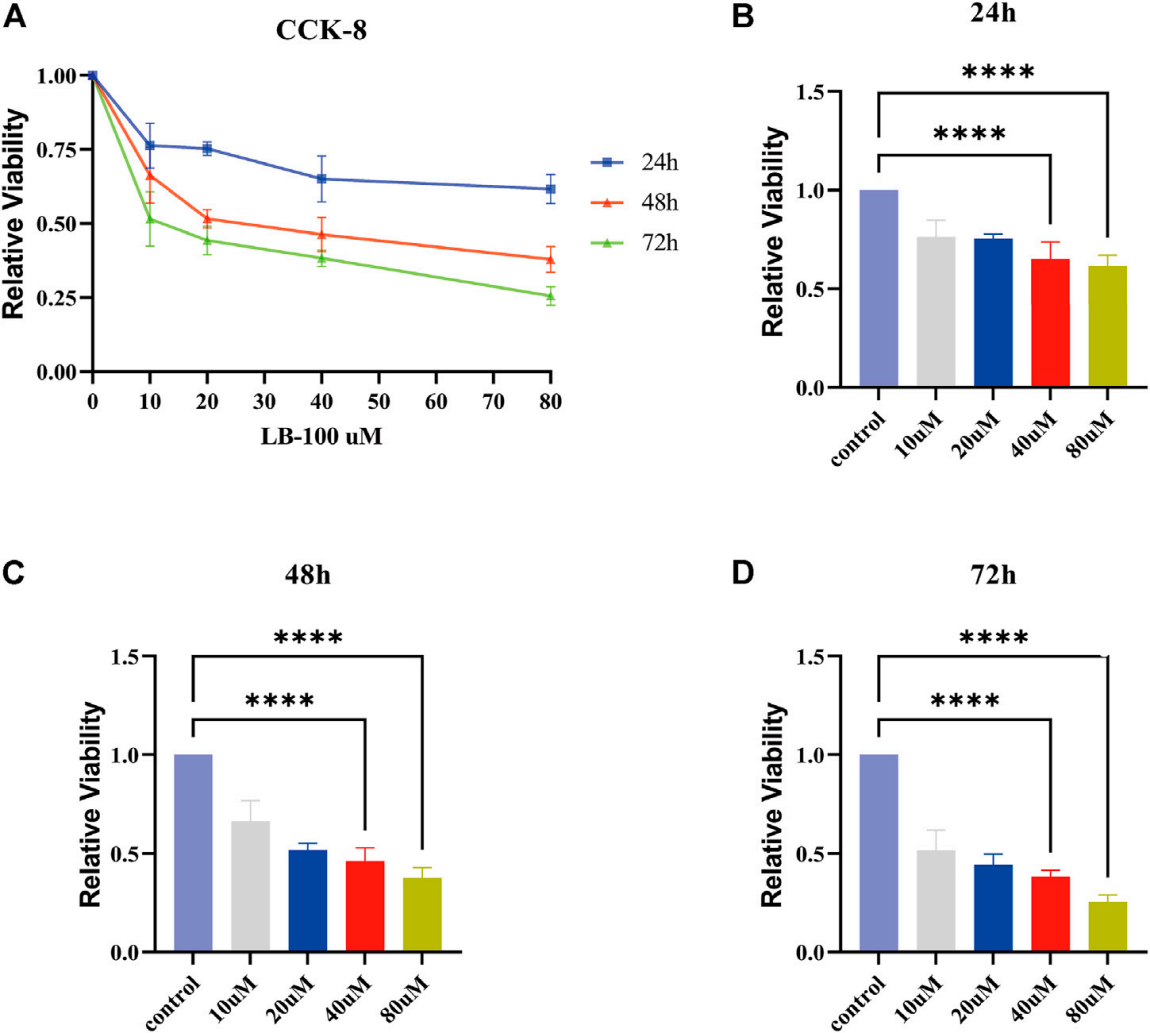
Effect of LB-100 on the proliferation of meningiomas. **(A)** The line chart displays the relative viability of IOMM-Lee cells treated with LB-100 at concentrations of 0 uM (control), 10, 20, 40, and 80 uM for 24, 48, and 72 h. The X axis represents the concentration of LB-100, and the Y axis represents relative viability. **(B–D)** The bar chart displays the relative viability for 24, 48, and 72 h at different concentrations of LB-100. The X-axis represents the concentration of LB-100, and the Y-axis represents relative viability; **p < 0.01, ****p < 0.0001.

For verification of the inhibitory effect of LB-100 on meningioma cell growth, the IOMM-Lee cell line was treated with different concentrations of LB-100 (10, 20, 40, and 80 uM) for different times (24, 48, and 72 h). The results showed that as the LB-100 treatment time increased, the relative activity of the cells decreased, which means that the inhibitory effect tended to increase. For the same treatment time, when the concentration of LB-100 increased, we observed that LB-100 significantly inhibited the growth of meningioma cells (Figure 4A). The results showed that compared with the control, LB-100 significantly inhibited the growth of IOMM-Lee at a concentration of 40 uM (*p* < 0.0001). Compared with the control, LB-100 also significantly inhibited the growth of IOMM-Lee at a concentration of 80 uM (*p* < 0.0001) (Figures 4B–D).

### LB-100 Increased the Phosphorylation of STAT1 (Tyr701) and the Expression of B7-H3

For determination of the effect of LB-100 on the phosphorylation of STAT1 and the expression of B7-H3, IOMM-Lee cells were treated with different concentrations of LB-100 (10, 20, 40, and 80 uM). The results showed that compared with that of the control group, the phosphorylation (Tyr701) of STAT1 was significantly increased at a concentration of 40 uM (*p* < 0.0001). Compared with that of the control group, the phosphorylation (Tyr701) of STAT1 was also significantly increased at a concentration of 80 uM (*p* < 0.0001). Therefore, we considered that when the concentration of LB-100 was 40 or 80 uM, the phosphorylation (Tyr701) of STAT1 increased significantly.

Compared with that of the control group, the expression of B7-H3 was significantly increased with 40 uM LB-100 treatment (*p* = 0.0396). The expression of B7-H3 was significantly increased in the 80 uM treatment group compared with the control group (*p* = 0.0277). Overall, the expression of B7-H3 increased significantly when the LB-100 concentration was 40 uM or 80 uM (Figure 3B).

## Discussion

Malignant meningiomas recur and have a dismal prognosis. Conventional treatment methods (including radiation, chemotherapy and targeted medical therapies) have not been effective in improving the survival rate of high-grade meningiomas [21]. One of the potential therapeutic strategies is to combine drugs that synergize with each other to maximize antitumor benefit at lower potential and lower toxic doses.

Proteomics can be used to conduct large-scale studies on all proteins expressed in tumors. Proteomics can not only effectively detect protein–protein interactions in tumor tissues and cells but also reveal the function and regulation of signaling pathways in tumors. However, the use of proteomics to systematically search for immune-related proteins and their regulatory pathways in malignant meningiomas has not yet been reported, which is of great significance in the study of malignant meningioma immunotherapy. Therefore, in this study, proteomic analysis was performed to discover immune-related proteins and their regulatory pathways in malignant meningiomas. The results of our proteomic study showed that the immune checkpoint protein B7-H3 was significantly overexpressed in malignant meningiomas compared with meningothelial meningiomas. In subsequent experiments, we further confirmed that B7-H3 was significantly overexpressed in malignant meningiomas. In 2018, Proctor et al. confirmed that B7-H3 is highly expressed in malignant meningiomas [22]. Our findings are consistent with previous studies.

At the same time, we performed proteomics and bioinformatics analyses to discover the STAT1/B7H3 immune checkpoint protein signaling pathway regulated by PP2A. Our proteomic results showed that compared with that in meningothelial meningiomas, the expression of STAT1 was increased in malignant meningiomas. STAT1 is an important part of the JAK/STAT pathway, and the JAK/STAT pathway is the central pathway of cytokine receptor signal transduction. It is crucial that the phosphorylation activation of STAT1 can regulate the expression of immune checkpoint proteins, including PD-L1 and B7-H3 [23, 24, 25]. However, there is no report on the expression of STAT1 and B7-H3 in meningioma. Our experiments showed that the PP2A inhibitor LB-100 can significantly increase the phosphorylation of STAT1 and the expression of B7-H3 in malignant meningiomas at the cellular level. Moreover, it can be inferred from our results that the PP2A inhibitor LB-100 may increase the expression of the immune checkpoint protein B7-H3 by increasing the phosphorylation of STAT1. However, Purvis et al. also found that the activation of STAT1 can downregulate the expression of B7H3 by decreasing MYC expression in medulloblastoma cells, which was different from our results. This discrepancy may be due to the regulation of STAT1 on the expression of B7-H3 depending on different molecular signaling mechanism in different tumors.

Many previous research results have shown that the protein phosphatase 2A (PP2A) inhibitor LB-100 has an obvious therapeutic effect on a variety of tumors by inhibiting the catalytic function of PP2A. The PP2A inhibitor LB-100 can reduce cell viability in salivary gland mucoepidermoid carcinoma and medulloblastoma [26, 27]. Studies on chordoma and glioblastoma show that LB-100 treatment increases G2/M arrest in tumor cells [28, 29]. Hu et al. proved that in secondary acute myeloid leukemia, the PP2A inhibitor LB-100 triggers G2/M arrest in tumor cells by regulating cell cycle regulatory proteins, and it can also induce apoptotic cell death [30]. This finding indicates that LB-100 may induce cytotoxicity by affecting the cell cycle and inducing apoptosis. Our results demonstrated that LB-100-mediated inhibition of meningioma cell viability was independent of its effect on upregulation of B7-H3 expression. LB-100 was cytotoxic to meningioma cells, and the inhibitory effect was positively correlated with the concentration of LB-100 and the time of LB100 treatment. In 2018, Ho et al. confirmed that the PP2A inhibitor LB-100 was cytotoxic to malignant meningioma cells [31], which was consistent with our experimental results.

Currently, there are many studies focusing on the combination therapy of LB-100 with other traditional treatments. Mirzapoiazova et al. proved that in small cell lung cancer (SCLC), the combined treatment of LB-100 and platinum-based chemotherapeutics can have a synergistic antitumor effect with low toxicity both *in vivo* and *in vitro* [32]. LB-100 also increases the sensitivity of meningiomas to radiotherapy [31]. Therefore, the combined treatment of LB-100 with radiotherapy or chemotherapy may have broad application prospects in the treatment of malignant meningioma. Moreover, the combined treatment of LB-100 with other immune checkpoint inhibitors has been shown to have significant therapeutic effects on a variety of malignant tumors. LB-100 increases the sensitivity of tumors to immune checkpoint inhibitors by upregulating the expression of immune checkpoint proteins [33]. Cui et al. proved that the PP2A inhibitor LB-100 can increase the expression of PD-L1 in glioblastoma both *in vivo* and *in vitro* [33]. Giles et al. proved that the combination of PP2A inhibitors and PD-L1 antibodies can promote immune-mediated antitumor activity and effectively kill meningiomas through antibody-dependent cell-mediated cytotoxicity (ADCC) [34]. Our study demonstrated that PP2A inhibitors can increase the expression of B7-H3 in malignant meningiomas; therefore, the combination of LB-100 and B7-H3 inhibitors may become a new and effective treatment method for malignant meningiomas.

In summary, our experimental results showed that the expression of B7-H3 is upregulated in malignant meningiomas and that the PP2A inhibitor LB-100 can significantly inhibit the proliferation and invasion of malignant meningioma cells while increasing the Tyr701 phosphorylation of STAT1 in malignant meningioma cells. These results showed that the PP2A inhibitor LB-100 has the potential to increase the sensitivity of malignant meningiomas to B7-H3-targeted immunotherapy.

## Data Availability

The original contributions presented in the study are included in the article/Supplementary Material, further inquiries can be directed to the corresponding author.
